# The Impact of Signet Ring Cell Differentiation on Outcome in Patients with Esophageal and Gastroesophageal Junction Adenocarcinoma

**DOI:** 10.1245/s10434-019-07322-x

**Published:** 2019-04-02

**Authors:** Sander J. M. van Hootegem, B. Mark Smithers, David C. Gotley, Sandra Brosda, Iain G. Thomson, Janine M. Thomas, Michael Gartside, Jan J. B. van Lanschot, Sjoerd M. Lagarde, Bas P. L. Wijnhoven, Andrew P. Barbour

**Affiliations:** 1 Department of Surgery, Erasmus MC, University Medical Center, Rotterdam, The Netherlands; 2 Upper Gastrointestinal/Soft Tissue Unit, Princess Alexandra Hospital, Brisbane, QLD Australia; 3 The University of Queensland, Brisbane, QLD Australia; 4 Mater Medical Research Institute, Mater Health Services, Raymond Terrace, South Brisbane, QLD Australia; 5 The University of Queensland, Diamantina Institute, Translational Research Institute, Woolloongabba, QLD Australia

## Abstract

**Background:**

Little is known about the association between signet ring cell (SRC) differentiation and response to neoadjuvant chemotherapy (nCT) or neoadjuvant chemoradiotherapy (nCRT) in patients with esophageal and junctional adenocarcinoma (EAC). We aimed to assess if SRC differentiation is associated with survival and response to nCT or nCRT in patients with EAC.

**Methods:**

Patients who underwent nCT and nCRT followed by surgery for EAC from 2000 until 2016 were identified from two institutional prospectively maintained databases. The pretreatment biopsy report or surgical resection specimen was used to differentiate patients into an SRC or non-SRC group.

**Results:**

Overall, 129 (19%) of 689 patients included had SRCs (nCT: *n* = 64; nCRT: *n* = 65). The SRC group had a more advanced ypT stage (*p* = 0.003), a higher number of positive lymph nodes in the resection specimen {median (interquartile range [IQR]) 2 [0–5] vs. 1 [0–3]; *p* = 0.002} and a higher rate of R1/R2 resections (19.4% vs. 12%; *p* = 0.026). SRC differentiation was not an independent prognostic factor for overall survival (OS) or disease-free survival (DFS). Following nCT, the SRC group had significantly shorter DFS (median [IQR] 12 [5–50] vs. 23 [8–164]; *p* = 0.013), but not OS, compared with the non-SRC group. In contrast, no differences according to SRC status for OS or DFS were found in patients who underwent nCRT.

**Conclusions:**

SRC differentiation was not independently associated with worse OS in patients with EAC who underwent neoadjuvant therapy and surgery. However, nCRT was associated with greater tumor downstaging and better DFS.

**Electronic supplementary material:**

The online version of this article (10.1245/s10434-019-07322-x) contains supplementary material, which is available to authorized users.

At present, neoadjuvant chemotherapy (nCT) or neoadjuvant chemoradiotherapy (nCRT) is indicated for patients with resectable esophageal cancer, given the improvement of survival in patients after multimodality treatment. However, the survival benefit of neoadjuvant treatment is limited to patients who respond to this treatment as defined by pathological response evaluation.^1^ A complete pathologic response is used as a prognostic indicator for overall survival (OS); however, a recent study questioned its validity as a surrogate endpoint for OS.^2^ Ideally, pretreatment genetic markers could serve as novel predictors of response to preoperative therapy. As there has been little progress in this field, histological subtyping might serve as an alternative.

Little is known about the effect of signet ring cell (SRC) differentiation on response to nCT or nCRT and survival in patients with esophageal and junctional adenocarcinoma (EAC). The optimal neoadjuvant treatment strategy for SRC tumors is therefore unknown. In patients with gastric cancer, SRCs are associated with poor prognosis.^3^ Recent studies have shown that response to nCT is poor in patients with SRC gastric cancer, and the benefit of nCT in this type of tumor is debated.^4^,^5^ SRC histology in patients with EAC may also be used as a predictor of prognosis and response to treatment. A pretreatment assessment of SRC differentiation in tumor biopsies could help to select patients who may or may not benefit from neoadjuvant treatment, and to predict prognosis in patients. Moreover, multimodality approaches are associated with a considerable rate of adverse events^6^^–^8 and it would be of great help to be able to better identify patients who benefit from preoperative treatment.

The rate of response to nCT in EAC patients with SRC tumors seems to be limited.^5^ However, Bekkar et al.^9^ found that patients with locally advanced SRC adenocarcinoma of the esophagus treated with chemoradiotherapy prior to surgery have a better outcome compared with surgery alone. Due to the local infiltrating character of SRC, chemoradiotherapy could potentially have a positive effect in these patients as adjunctive locoregional treatment prior to surgery.^4^ In their study, Bleaney et al.^10^ concluded that patients with adenocarcinomas with SRC differentiation have a different response to neoadjuvant therapy than non-SRC adenocarcinomas, but the exact impact of SRC differentiation on neoadjuvant treatment and prognosis in esophageal adenocarcinomas remains unclear. Therefore, the aim of this study was to investigate the association of SRC differentiation with response to neoadjuvant treatment and survival in patients with EACs.

## Methods

### Patients

A retrospective study was conducted using two prospectively maintained databases of the Departments of Surgery at the Erasmus University Medical Centre (Erasmus MC), Rotterdam, The Netherlands, and the Princess Alexandra Hospital (PA Hospital), Brisbane, Australia. In both institutions, ethical approval was obtained. All patients who underwent nCT or nCRT followed by esophagectomy with curative intent for tumors of the gastroesophageal junction or esophagus from 2000 until 2016 were included. Patients needed to have completed at least two cycles of chemotherapy and, if applicable, received a minimum total radiation dose of 35 Gy. Patient records were reviewed to obtain information when missing. Pretreatment biopsy reports were used to assess the tumor histology and to differentiate patients into an SRC or non-SRC group. In some patients, pretreatment biopsy reports were not available. In these cases, the surgical resection specimen was used to determine whether the tumor showed SRC differentiation or not. A tumor was classified as having SRC differentiation when any SRC morphology was seen in the histologically assessed tissue, independent of the percentage. Complete pathological responders who did not have a biopsy report available were excluded. The non-SRC group served as a reference group.

### Pretreatment Staging

All patients were staged by endoscopy and computed tomography (CT) of the chest and abdomen. Endoscopic ultrasonography was used in selected patients from the PA Hospital to clarify tumor and nodal staging, whereas it was routinely used in all Erasmus MC patients. In the PA Hospital, fluorodeoxyglucose-positron emission tomography (FDG-PET) scanning has been routinely performed since 2008, and, in the Erasmus MC, FDG-PET scanning was introduced in 2008 to obtain assurance of no further distant dissemination when conventional imaging showed signs of extensive lymph node involvement and became a standard procedure in 2013.

### Treatment

The nCRT regimen administered to all patients from the Erasmus MC was as per the CROSS protocol.^11^ PA Hospital patients mainly received a combination of two cycles cisplatin and 5-fluoruracil administered with a total radiation dose of either 35 Gy in 15 fractions or 45 Gy in 25 fractions, commencing the radiotherapy with the second cycle of chemotherapy. A small number of these patients were administered additional docetaxel. Since 2015, there has been an increasing use of the CROSS regimen in PA Hospital patients. The majority of the Erasmus MC nCT patients was treated with either carboplatin or cisplatin, in combination with paclitaxel. Other patients received perioperative chemotherapy consisting of a combination of epirubicin, cisplatin and capecitabine, administered in three cycles before surgery and three cycles after surgery. PA Hospital patients receiving nCT were administered similar chemotherapeutic regimens as their nCRT-treated patients, most commonly as per the OEO2 protocol.^12^ However, a moderate number of nCT patients were treated according to the MAGIC protocol.^13^ The surgical technique used was dependent on tumor location and local expertise or preferences. Details of the surgical techniques in the PA Hospital and Erasmus MC have been previously described.^14^,^15^

### Pathological Assessment

All resection specimens were assessed by experienced gastrointestinal (GI) pathologists to determine the pathologic tumor (ypT), nodal (ypN) and distant metastasis (ypM) stage in accordance with the TNM staging system of the Union for International Cancer Control/American Joint Committee on Cancer (7th edition).^16^ Specimens with tumor cells present within 1 mm of the resection margin were considered to be an R1 resection.^17^ Tumor regression was graded according to the Mandard score.^18^

### Follow-Up and Recurrence

After esophagectomy, patients were seen every 3 months for the first 2 years. The following 3 years, patients were assessed at 6-month intervals, and annually up to 5 (Erasmus MC) or 10 years (PA Hospital). Follow-up visits included patient’s history and physical examination. Symptoms suggestive of recurrence were investigated using a CT scan and endoscopy if indicated. Further investigations were performed on individual basis. Recurrence was documented by site of first recurrence, dividing it into locoregional, distant, or both. Locoregional recurrence was defined as disease recurring within the previous esophageal bed, at the anastomotic site, or as disease recurring in the draining lymphatic basins, depending on the prior tumor site. Distant recurrence was defined as any lymphatic dissemination further than regional lymphatic basins, as well as recurrence in any distant organ. Recurrence present in more than one anatomical location was regarded as synchronous if detected within 4 weeks of documented recurrence.

### Statistical Analysis

Differences between groups were tested using Pearson’s Chi square test or Fisher’s exact test for categorical data, and Mann–Whitney U test for non-parametric continuous data. Categorical variables were reported as numbers and percentages, and distribution of continuous characteristics was reported as median (interquartile range [IQR]) or mean ± standard deviation (SD). OS was calculated as the time between surgery and death by any cause or last follow-up, while disease-free survival (DFS) was calculated as the time between surgery and histologically proven or radiological evidence of recurrence, or death by any cause. Survival curves were obtained using the Kaplan–Meier method, and differences were tested using the log-rank test. Cox regression analysis was used to assess the relation of clinical and pathological variables with OS and DFS. Multiple multivariable models were composed to assess the prognostic significance of SRC differentiation on OS and DFS, separately for nCT and nCRT. A *p* value ≤ 0.05 (two-sided) was considered to be statistically significant for all data. All analysis was performed using SPSS^®^ version 25.0 (IBM Corporation, Armonk, NY, USA).

## Results

### Patients

A total of 714 patients matched the inclusion criteria. Twenty-five patients were excluded as detailed pathology reports were missing. Of the remaining 689 study patients, 129 patients had tumors that showed SRC histology (nCT, *n* = 64; nCRT, *n* = 65), and 560 patients had no evidence of SRC (nCT, *n* = 234; nCRT, *n* = 326). A total of 93 (SRC, *n* = 10; non-SRC, *n* = 83) patients did not have a biopsy report available and the resection specimen was used to determine whether the tumor showed SRC histology. Electronic supplementary Table 1 shows details regarding neoadjuvant treatment.

No statistically significant differences in clinical characteristics were found between the SRC and non-SRC groups (Table 1); however, pathological T stage (ypT stage) [*p* = 0.003], number of positive lymph nodes in the resection specimen (median [IQR] 2 [0–5] vs. 1 [0–3]; *p* = 0.002) and proportion of R1/R2 resections (19.4% vs. 12.0%; *p* = 0.026) were higher in the SRC group (Table 2). No significant difference was seen in response to therapy according to the Mandard score, between the SRC and non-SRC groups. A comparison between Erasmus MC and PA Hospital patients is shown in electronic supplementary Table 2.

**Table 1:** Clinical and tumor characteristics according to the presence of SRC

Variables	SRC [*n* = 129]	Non-SRC [*n* = 560]	*p* value
Age, years (mean [SD])	61.98 [8.44]	61.53 [9.07]	0.945
Sex			
Male	117 (90.7)	500 (89.3)	0.636
Female	12 (9.3)	60 (10.7)	
Tumor location			
Upper esophagus	–	2 (0.4)	0.596
Middle	4 (3.1)	30 (5.4)	
Lower	70 (54.3)	309 (55.2)	
GO junction	55 (42.6)	219 (39.1)	
cT stage			
T1	–	12 (2.1)	0.213
T2	30 (23.3)	146 (26.1)	
T3	92 (71.3)	373 (66.6)	
T4	3 (2.3)	21 (3.8)	
Missing	4 (3.1)	8 (1.4)	
cN stage			
N0	60 (46.5)	253 (45.2)	0.373
N1	50 (38.8)	219 (39.1)	
N2	10 (7.8)	68 (12.1)	
N3	2 (1.6)	8 (1.4)	
N+	1 (0.8)	1 (0.2)	
Nx	2 (1.6)	4 (0.7)	
Missing	4 (3.1)	7 (1.3)	
cM stage			
M0	107 (82.9)	475 (84.8)	0.503
M1	7 (5.4)	28 (5)	
Mx	11 (8.5)	50 (8.9)	
Missing	4 (3.1)	7 (1.3)	
Neoadjuvant treatment			
nCRT	65 (50.4)	326 (58.2)	0.106
nCT	64 (49.6)	234 (41.8)	

Data are expressed as *n* (%) unless otherwise specified

*SRC* signet ring cell, *SD* standard deviation, *GO* gastroesophageal, *nCRT* neoadjuvant chemoradiotherapy, *nCT* neoadjuvant chemotherapy

**Table 2:** Pathological characteristics according to the presence of SRC

Variable	SRC [*n* = 129]	Non-SRC [*n* = 560]	*p* value
ypT stage			
T0	8 (6.2)	6 (11.2)	**0.003**
Tis	–	2 (0.4)	
T1	10 (7.8)	110 (19.6)	
T2	21 (16.3)	102 (18.2)	
T3	87 (67.4)	266 (47.5)	
T4	3 (2.3)	17 (3)	
Missing	–	1 (0.2)	
ypN stage			
N0	50 (38.8)	256 (45.7)	0.323
N1	37 (28.7)	168 (30)	
N2	23 (17.8)	80 (14.3)	
N3	19 (14.7)	55 (9.8)	
Missing	–	1 (0.2)	
ypM stage			
M0	117 (90.7)	535 (95.5)	0.126
M1	11 (8.5)	23 (4.1)	
Mx	1 (0.8)	1 (0.2)	
Missing	–	1 (0.2)	
Mandard			
TRG 1–2	33 (25.6)	183 (32.7)	0.196
TRG 3–5	83 (64.3)	312 (55.7)	
Missing	13 (10.1)	65 (11.6)	
Resection margin			
R0	104 (80.6)	493 (88)	**0.026**
R1/R2	25 (19.4)	67 (12)	
Lymph node yield (median [IQR])	20 [16–26]	20 [15–28]	0.655
Positive lymph nodes (median [IQR])	2 [0–5]	1 [0–3]	**0.002**

Data are expressed as *n* (%) unless otherwise specified

Bold values indicate statistical significance (*p* < 0.05; two-sided)

*SRC* signet ring cell, *TRG* tumor regression grade, *IQR* interquartile range

The subgroup of patients with SRC tumors treated with nCT had more advanced ypT stage (*p* = 0.004) and more irradical resections (29.7% vs. 17.9; *p* = 0.039). There were no statistically significant differences in pathological characteristics between the SRC and non-SRC groups for patients treated with nCRT (electronic supplementary Table 3).

### Overall Survival

**Fig. 1:**
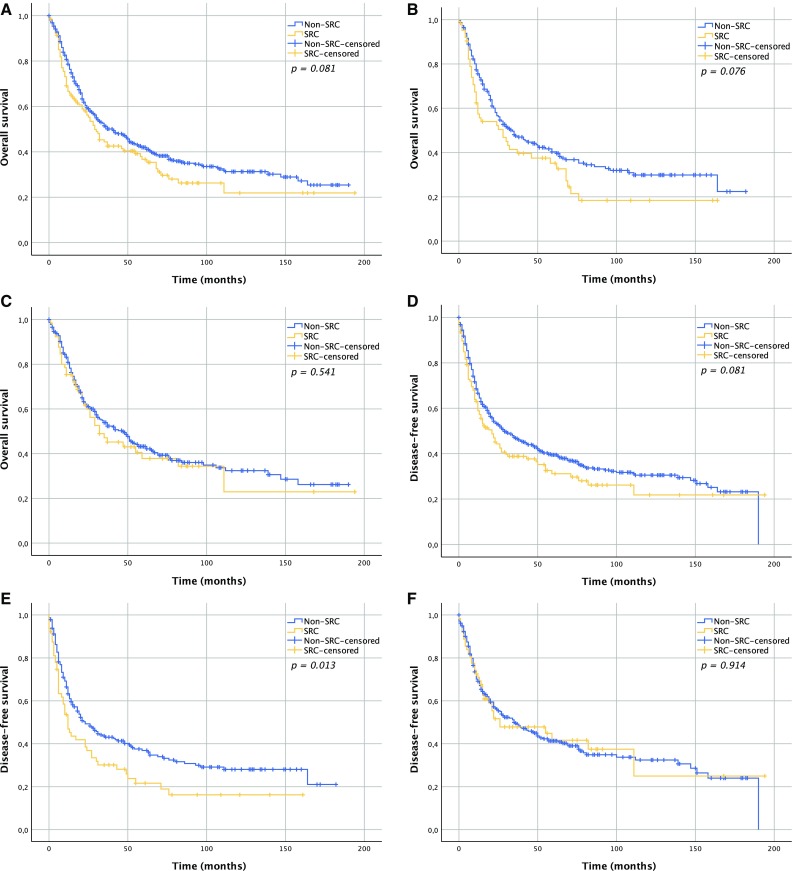

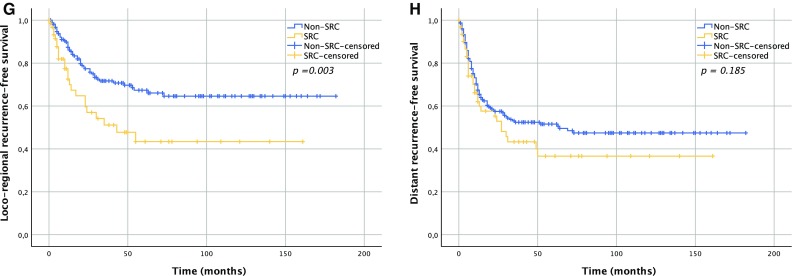
Survival curves according to SRC status. **a** Overall survival; *p* = 0.081; **b** overall survival in the nCT group; *p* = 0.076; **c** overall survival in the nCRT group; *p* = 0.541; **d** disease-free survival; *p* = 0.081; **e** disease-free survival in the nCT group; *p* = 0.013; **f** disease-free survival in the nCRT group; *p* = 0.914; **g** locoregional recurrence-free survival in the nCT group; *p* = 0.003; **h** distant recurrence-free survival in the nCT group; *p* = 0.185. *SRC* signet ring cell, *nCRT* neoadjuvant chemoradiotherapy, *nCT* neoadjuvant chemotherapy

Median OS time for the SRC group was 29 months (IQR 10–111), whereas non-SRC patients had a median OS of 41 months (IQR 14–not reached; *p* = 0.081) (Fig. 1a). There was also no statistically significant difference in OS according to SRC status following nCT (*p* = 0.076) (Fig. 1b) or nCRT (*p* = 0.541) (Fig. 1c).

In nCT patients, only advanced ypTNM stage, R1/R2 resection, and SRC status were associated with worse OS in univariable analysis (Table 3). However, in multivariable analysis, SRC status was not an independent predictor of OS, while resection margin remained a significant predictor. Univariable analysis in nCRT patients showed that increased age, advanced ypTNM stage, Mandard score of 3–5, and an R1/R2 resection status were associated with lower OS. In multivariable analysis, age and advanced ypTNM stage were independent predictors of OS. SRC status was not associated with worse OS, neither in univariable nor multivariable analysis.

**Table 3:** Univariable and multivariable analysis of the effects of clinicopathological variables on OS

Variables	nCT					nCRT				
	*n*	Univariable analysis		Multivariable analysis		*n*	Univariable analysis		Multivariable analysis	
		HR (95% CI)	*p* value	HR (95% CI)	*p* value		HR (95% CI)	*p* value	HR (95% CI)	*p* value
Age	298	0.997 (0.98–1.01)	0.71			391	1.02 (1.00–1.03)	0.06	1.02 (1.01–1.04)	0.004
Sex										
Male	265	Reference				352	Reference		Reference	
Female	33	0.92 (0.57–1.48)	0.72			39	0.64 (0.39–1.07)	0.09	0.68 (0.41–1.14)	0.15
Tumor location										
Upper/middle esophagus	11	Reference				25	Reference			
Lower	150	0.78 (0.38–1.61)	0.50			229	0.97 (0.58–1.63)	0.90		
GO junction	137	0.73 (0.36–1.52)	0.41			137	0.83 (0.48–1.43)	0.51		
ypT stage										
T0	14	Reference		Reference		56	Reference		Reference	
T1	46	0.60 (0.24–1.50)	0.27	0.53 (0.21–1.34)	0.18	74	1.73 (0.96–3.12)	0.07	1.74 (0.95–3.19)	0.08
T2	46	0.72 (0.29–1.79)	0.48	0.50 (0.20–1.26)	0.14	77	1.96 (1.10–3.49)	0.02	1.56 (0.84–2.91)	0.16
T3	177	1.57 (0.69–3.57)	0.29	0.61 (0.26–1.47)	0.27	176	3.13 (1.85–5.28)	< 0.001	2.19 (1.20–4.01)	0.01
T4	15	3.4 (1.28–9.01)	0.01	1.09 (0.39–3.10)	0.87	5	8.47 (2.80–25.60)	< 0.001	5.46 (1.51–19.71)	0.01
ypN stage										
N0	94	Reference		Reference		212	Reference		Reference	
N1	95	2.75 (1.82–4.17)	< 0.001	2.48 (1.62–3.79)	< 0.001	120	2.13 (1.56–2.91)	< 0.001	2.12 (1.51–3.00)	< 0.001
N2	62	4.08 (2.61–6.37)	< 0.001	4.18 (2.59–6.75)	< 0.001	41	3.49 (2.33–5.25)	< 0.001	2.96 (1.86–4.72)	< 0.001
N3	47	5.94 (3.72–9.48)	< 0.001	5.17 (3.11–8.60)	< 0.001	27	4.52 (2.87–7.11)	< 0.001	3.70 (2.23–6.15)	< 0.001
ypM stage										
M0	277	Reference		Reference		375	Reference		Reference	
M1	21	4.16 (2.59–6.68)	< 0.001	4.04 (2.42–6.73)	< 0.001	13	3.46 (1.92–6.23)	< 0.001	3.13 (1.65–5.92)	< 0.001
Mandard										
TRG 1–2	35	Reference				181	Reference		Reference	
TRG 3–5	232	0.56 (0.21–1.52)	0.26			198	1.61 (1.23–2.12)	0.001	0.85 (0.61–1.19)	0.34
Resection margin										
R0	247	Reference		Reference		360	Reference		Reference	
R1/R2	61	3.09 (2.23–4.27)	< 0.001	1.93 (1.34–2.79)	< 0.001	31	1.84 (1.18–2.86)	0.007	1.04 (0.62–1.73)	0.77
SRC status										
Non-SRC	234	Reference		Reference		326	Reference		Reference	
SRC	64	1.36 (0.97–1.90)	0.08	1.08 (0.76–1.54)	0.66	65	1.12 (0.79–1.58)	0.54	0.86 (0.59–1.24)	0.41

Variables were entered in the multivariable model if the *p* value was < 0.1 in the univariable analysis

The Mx (*n* = 2) and Tis (*n* = 2) categories were not included in the analysis

*nCRT* neoadjuvant chemoradiotherapy *nCT* neoadjuvant chemotherapy, *HR* hazard ratio, *CI* confidence interval, *GO* gastroesophageal, *TRG* tumor regression grade, *SRC* signet ring cell, *OS* overall survival

### Disease-Free Survival

Median DFS for SRC and non-SRC patients was 21 months (IQR 6–111) and 29 months (IQR 9–164), respectively (*p* = 0.081) (Fig. 1d). For patients who underwent nCT, the SRC group had a median DFS of 12 months (IQR 5–50) compared with 23 months (IQR 8–164) for the non-SRC group (*p* = 0.013) (Fig. 1e). In patients who underwent nCRT, median DFS was 26 months (IQR 10–111) in the SRC group, and 35 months (IQR 10–158) in the non-SRC group (*p* = 0.914) (Fig. 1f). nCT patients in the SRC group had worse locoregional recurrence-free survival (RFS; *p* = 0.003) (Fig. 1g), but not distant RFS (*p* = 0.185) (Fig. 1h). Multivariable analysis showed that advanced ypN and ypM staging were independent predictors of DFS in both nCRT and nCT patients (Table 4). Among nCT patients, resection margin was also a significant predictor of DFS, in contrast to SRC status, which was not.

**Table 4:** Univariable and multivariable analysis of effects of clinicopathological variables on DFS

Variables	nCT					nCRT				
	*n*	Univariable analysis		Multivariable analysis		*n*	Univariable analysis		Multivariable analysis	
		HR (95% CI)	*p* value	HR (95% CI)	*p* value		HR (95% CI)	*p* value	HR (95% CI)	*p* value
Age	298	1.00 (0.98–1.02)	0.91			391	1.01 (1.00–1.03)	0.11		
Sex										
Male	265	Reference				352	Reference			
Female	33	0.92 (0.59–1.46)	0.73			39	0.74 (0.46–1.20)	0.22		
Tumor location										
Upper/middle esophagus	11	Reference				25	Reference			
Lower	150	0.90 (0.44–1.84)	0.76			229	1.00 (0.59–1.68)	0.99		
GO junction	137	0.86 (0.42–.78)	0.69			137	0.90 (0.52–1.56)	0.71		
ypT stage										
T0	14	Reference		Reference		56	Reference		Reference	
T1	46	0.74 (0.30–1.83)	0.51	0.66 (0.26–1.65)	0.37	74	1.74 (0.98–3.09)	0.06	1.77 (0.98–3.20)	0.06
T2	46	0.85 (0.34–2.10)	0.72	0.57 (0.22–1.65)	0.23	77	1.97 (1.12–3.45)	0.02	1.52 (0.83–2.77)	0.18
T3	177	1.73 (0.76–3.95)	0.19	0.60 (0.25–1.44)	0.25	176	3.00 (1.80–5.00)	< 0.001	2.15 (1.19–3.90)	0.01
T4	15	3.52 (1.33–9.32)	0.01	1.12 (0.395–3.18)	0.83	5	6.36 (2.13–19.05)	0.001	4.19 (1.19–14.68)	0.03
ypN stage										
N0	94	Reference		Reference		212	Reference		Reference	
N1	95	2.85 (1.90–4.30)	< 0.001	2.59 (1.70–3.95)	< 0.001	120	2.40 (1.77–3.26)	< 0.001	2.36 (1.69–3.31)	< 0.001
N2	62	4.71 (3.06–7.26)	< 0.001	4.98 (3.11–8.00)	< 0.001	41	3.13 (2.06–4.76)	< 0.001	2.57 (1.61–4.10)	< 0.001
N3	47	5.88 (3.72–9.29)	< 0.001	5.42 (3.29–8.94)	< 0.001	27	4.35 (2.71–6.99)	< 0.001	3.27 (1.92–5.57)	< 0.001
ypM stage										
M0	277	Reference		Reference		375	Reference		Reference	
M1	21	5.10 (3.20–8.14)	< 0.001	5.48 (3.32–9.04)	< 0.001	13	4.16 (2.36–7.33)	< 0.001	4.30 (2.26–8.18)	< 0.001
Mandard										
TRG 1–2	35	Reference				181	Reference		Reference	
TRG 3–5	232	0.61 (0.23–1.65)	0.33			198	2.28 (1.34–3.88)	0.002	0.85 (0.60–1.19)	0.33
Resection margin										
R0	247	Reference		Reference		360	Reference		Reference	
R1/R2	61	3.01 (2.18–4.14)	< 0.001	1.97 (1.38–2.81)	< 0.001	31	1.73 (1.10–2.72)	0.02	0.96 (0.56–1.63)	0.87
SRC status										
Non-SRC	234	Reference		Reference		326	Reference		Reference	
SRC	64	1.50 (1.08–2.07)	0.02	1.27 (0.90–1.78)	0.17	65	0.98 (0.68–1.41)	0.92	0.76 (0.51–1.12)	0.17

Variables were entered in the multivariable model if the *p* value was < 0.1 in the univariable analysis

The Mx (*n* = 2) and Tis (*n* = 2) categories were not included in the analysis

*nCRT* neoadjuvant chemoradiotherapy, *nCT* neoadjuvant chemotherapy, *HR* hazard ratio, *CI* confidence interval, *GO* gastroesophageal, *TRG* tumor regression grade, *SRC* signet ring cell, *DFS* disease-free survival

## Discussion

This study shows that in patients with EAC treated with neoadjuvant therapy plus surgery, SRC differentiation was not an independent predictor for OS or DFS. However, in patients who underwent nCT, SRC differentiation was associated with a higher rate of R1/R2 resections and worse locoregional RFS. Multivariable analysis in nCT patients showed that resection margin was an independent predictor of both OS and DFS.

Other studies have shown that tumors with SRC differentiation possess unique clinical features, but still little is known about the optimal treatment strategy for these tumors.^10^ Median survival of SRC patients was inferior to non-SRC patients. However, current nCRT treatment seems to lead to comparable outcomes in tumors that show SRC differentiation compared with tumors that do not. Until 2015, nCT was the standard preoperative therapy for EAC patients treated in the PA Hospital, while the role of radiotherapy is still debated.^19^ The results of this study raise the question as to whether the same outcome applies to SRC tumors.

With regard to nCT, it is possible that the higher number of R1/R2 resections in the SRC group could be a result of selection bias instead of more aggressive tumor behavior; however, our data indicate that this difference is not seen in patients treated with nCRT. In the present study, the percentage of radical (R0) resections after nCRT was comparable with previously reported data by Bekkar et al.^9^ In line with their study, we found that nCRT can lead to more favorable outcomes in SRC patients. In addition, lower radical resection rates in SRC patients treated with nCT were also reported in other recent literature,^2^,^4^ and resection margin status was an independent prognostic factor in nCT patients in our study. This underlines the prognostic impact of positive resection margins.

Ideally, all SRC-positive biopsies would have been reassessed by an experienced pathologist to differentiate the tumors into ≥ 50% and < 50% SRC groups. Unfortunately, this was not feasible due to the retrospective nature of the study. The majority of preoperative biopsies were performed in the referring hospitals before patients were sent to the tertiary center for (surgical) treatment. The resection specimens from the Rotterdam cohort were analyzed by two experienced GI pathologists, and the Brisbane samples were analyzed by experienced GI pathologists working at either the PA Hospital or one of two large private pathology practices. While blinded, central pathology review of all cases would be ideal, this was not possible for the present study as many patients’ tissue blocks or slides were unavailable. Clarification of the amount of SRC found in the biopsied tissue is said to be inaccurate to determine whether the tumor truly consists of 50% SRC or more.^20^ In addition, Patel et al.^21^ reported that mixed subtypes of adenocarcinoma, i.e. tumors consisting of a non-SRC component admixed with an SRC component, have inferior survival outcomes. In their study, SRC histology was a significant predictor of survival and thus they suggested that even a small percentage of SRCs can have a clinical impact on tumor behavior. This finding partly concurs with our study as we also found inferior survival times in the SRC group, although this did not reach statistical significance.

A limitation of this study is its retrospective nature, with inclusion of patients treated over an extended period of time, resulting in bias as a consequence of evolving treatment practices. However, selecting patients from two different centers limits the effect of possible selection bias and treatment habits. Furthermore, a variety of platinum-based regimens has been used due to the diverse standard treatments in both institutions. However, the vast majority of patients were treated with well-known and widely applied regimens according to the CROSS,^11^ MAGIC,^13^ and OEO2 trials.^12^ Finally, the resection specimens were analyzed by different pathologists, which might have affected uniformity in the assessment of response to therapy. To minimize the impact of possible interobserver variability, we grouped the Mandard scores into responder (tumor regression grade [TRG] 1–2) and non-responder (TRG 3–5) groups.

## Conclusions

SRC differentiation is not an independent predictor of OS or DFS in patients who underwent neoadjuvant therapy followed by surgery; however, SRC tumors may respond differently according to the type of neoadjuvant treatment. In nCT patients, the SRC group had more R1/R2 resections, which in turn was associated with worse locoregional RFS. Hence, it is possible that nCRT provides additional benefit in SRC patients as it could offer better locoregional control. Further study of the efficacy of locoregional therapy intensification would therefore seem warranted. Although this study brings nuance to the question as to what the impact is of SRC on neoadjuvant treatment, determining an optimal treatment strategy for EAC SRC tumors would require a multicenter cohort study comparing nCRT and nCT more directly.

## Electronic Supplementary Material

Below is the link to the electronic supplementary material.

Supplementary material 1 (DOCX 37 kb)
