# Supplementary material for: The Impact of Signet Ring Cell Differentiation on Outcome in Patients with Esophageal and Gastroesophageal Junction Adenocarcinoma
Source: Ann Surg Oncol. 2019 Apr 2;26(8):2375–84. doi: 10.1245/s10434-019-07322-x (PMC6611740; doi:10.1245/s10434-019-07322-x)
Supplement: Supplementary file 1 — Supplementary material 1 (DOCX 37 kb) [file 10434_2019_7322_MOESM1_ESM.docx]

**Supplement**

| **Supplementary table 1** Neoadjuvant treatments details | | | |
| --- | --- | --- | --- |
| **Neoadjuvant treatment** | |  | **n** |
| **nCRT** |  |  | **391** |
| *Carboplatin/Paclitaxel (CROSS)* | | | 242 |
| *Cisplatin/5FU* | |  | 128 |
| *Cisplatin/5FU/Docetaxel* | |  | 16 |
| *Other* |  |  | 3 |
| *Unknown* |  |  | 2 |
| **nCT** |  |  | **298** |
| *Cisplatin/5FU* | |  | 128 |
| *Epirubicin/Cisplatin/5FU* | |  | 48 |
| *Carboplatin/Paclitaxel* | |  | 40 |
| *Epirubicin/Cisplatin/Capecitabin* | | | 37 |
| *Cisplatin/Paclitaxel* | |  | 25 |
| *Cisplatin/5FU/Docetaxel* | |  | 16 |
| *Unknown* |  |  | 3 |
| *5FU* |  |  | 1 |

*5FU* 5-fluoruracil

| **Supplementary** t**able 2** Comparison Australian (PAH) versus Dutch (EMC) patients | | | | |  |  |
| --- | --- | --- | --- | --- | --- | --- |
|  |  |  |  | **PAH (%)** | **EMC (%)** |  |
|  |  |  |  | **n = 358** | **n = 331** | ***p-value*** |
| **Age, mean [SD]** | |  |  | 61.06 [8.88] | 62.21 [9.00] | 0.176 |
| **Gender** |  |  |  |  |  | 0.395 |
| Male |  |  |  | 324 (90.5) | 293 (88.5) |  |
| Female |  |  |  | 34 (9.5) | 38 (11.5) |  |
| **Location tumour** | |  |  |  |  | 0.118 |
| Upper/Middle Oesophagus |  |  |  | 13 (3.6) | 23 (6.9) |  |
| Lower |  |  |  | 196 (54.7) | 183 (55.3) |  |
| GO Junction |  |  |  | 149 (41.6) | 125 (37.8) |  |
| **Tumour differentiation** | |  |  |  |  | 0.431 |
| SRC |  |  |  | 63 (17.6) | 66 (19.9) |  |
| Non-SRC |  |  |  | 295 (82.4) | 265 (80.1) |  |
| **Neo. Treatment** | |  |  |  |  | **< 0.001** |
| nCRT |  |  |  | 165 (46.1) | 226 (68.3) |  |
| nCT |  |  |  | 193 (53.9) | 105 (31.7) |  |
| **cT** |  |  |  |  |  | **< 0.001** |
| T1 |  |  |  | 10 (2.8) | 2 (0.6) |  |
| T2 |  |  |  | 136 (38) | 40 (12.1) |  |
| T3 |  |  |  | 212 (59.2) | 253 (76.4) |  |
| T4 |  |  |  | - | 24 (7.3) |  |
| Missing |  |  |  | 10 (2.8) | 12 (3.6) |  |
| **cN** |  |  |  |  |  | **< 0.001** |
| N0 |  |  |  | 225 (62.8) | 88 (26.6) |  |
| N1 |  |  |  | 123 (34.4) | 146 (44.1) |  |
| N2 |  |  |  | 9 (2.5) | 69 (20.8) |  |
| N3 |  |  |  | 1 (0.3) | 9 (2.7) |  |
| N+ |  |  |  | - | 2 (0.6) |  |
| Nx |  |  |  | - | 6 (1.8) |  |
| Missing |  |  |  | - | 11 (3.3) |  |
| **cM** |  |  |  |  |  |  |
| M0 |  |  |  | 358 (100) | 224 (67.7) |  |
| M1 |  |  |  | - | 35 (10.6) |  |
| Mx |  |  |  | - | 61 (18.4) |  |
| Missing |  |  |  | - | 11 (3.3) |  |
| **Mandard** |  |  |  |  |  | **< 0.001** |
| TRG1 |  |  |  | 40 (11.2) | 34 (10.3) |  |
| TRG2 |  |  |  | 73 (20.4) | 69 (20.8) |  |
| TRG3 |  |  |  | 76 (21.2) | 82 (24.8) |  |
| TRG4 |  |  |  | 100 (27.9) | 65 (19.6) |  |
| TRG5 |  |  |  | 58 (16.2) | 14 (4.2) |  |
| Missing |  |  |  | 11 (3.1) | 67 (20.2) |  |
| **ypT** |  |  |  |  |  | **<0.001** |
| T0 |  |  |  | 37 (10.3) | 33 (10) |  |
| Tis |  |  |  | 2 (0.6) | - |  |
| T1 |  |  |  | 67 (18.7) | 53 (16) |  |
| T2 |  |  |  | 53 (14.8) | 70 (21.1) |  |
| T3 |  |  |  | 179 (50) | 174 (52.6) |  |
| T4 |  |  |  | 19 (5.3) | 1 (0.3) |  |
| Missing |  |  |  | 1 (0.3) | - |  |
| **ypN** |  |  |  |  |  | **< 0.001** |
| N0 |  |  |  | 143 (39.9) | 163 (49.2) |  |
| N1 |  |  |  | 94 (26.3) | 111 (35.5) |  |
| N2 |  |  |  | 73 (20.4) | 30 (9.1) |  |
| N3 |  |  |  | 47 (13.1) | 27 (8.2) |  |
| Missing |  |  |  | 1 (0.3) | - |  |
| **ypM** |  |  |  |  |  | **<0.001** |
| M0 |  |  |  | 350 (97.8) | 302 (91.2) |  |
| M1 |  |  |  | 7 (2) | 27 (8.2) |  |
| Mx |  |  |  | - | 2 (0.6) |  |
| Missing |  |  |  | 1 (0.3) | - |  |
| **Resection margin** | |  |  |  |  | 0.686 |
| R0 |  |  |  | 312 (87.2) | 285 (86.1) |  |
| R1/R2 |  |  |  | 46 (12.8) | 46 (13.9) |  |
| **Lymph node yield, median [IQR]** | | |  | 22 [17 - 30] | 18 [13 - 24.5] | **< 0.001** |
| **Positive lymph nodes, median [IQR]** | | |  | 1 [0 - 4] | 1 [0 - 2] | **0.006** |
| *PAH* Princess Alexandra Hospital, *EMC* Erasmus MC, *SD* standard deviation, *GO* gastro-oesophageal, *SRC* signet ring cell, *nCRT* neoadjuvant chemoradiotherapy, *nCT* neoadjuvant chemotherapy, *IQR* interquartile range; **bold** values are statistically significant (*p* < 0.05; two-sided) | | | | | | |

| **Supplementary table 3** Pathological characteristics of nCRT and nCT patients (SR vs non-SRC) | | | | | | | | | | | | | | | |  |  |
| --- | --- | --- | --- | --- | --- | --- | --- | --- | --- | --- | --- | --- | --- | --- | --- | --- | --- |
|  | | | | |  | **nCRT** | |  | | | |  | |  | **nCT** |  |  |
|  | | | | |  | **n = 391** | |  | | | |  | |  | **n = 298** |  |  |
|  | | | | |  | **SRC (%)** | | **Non-SRC (%)** | | | |  | |  | **SRC (%)** | **Non-SRC (%)** |  |
|  | | | | |  | **n = 65** | | **n = 326** | | | | ***p-value*** | |  | **n = 64** | **n = 234** | ***p-value*** |
| **ypT stage** | | | | |  |  | |  | | | | 0.299 | |  |  |  | **0.004** |
| T0 | | | | |  | 7 (10.8) | | 49 (15) | | | |  | |  | 1 (1.6) | 13 (5.6) |  |
| Tis | | | | |  | - | | 2 (0.6) | | | |  | |  | - | - |  |
| T1 | | | | |  | 7 (10.8) | | 67 (20.6) | | | |  | |  | 3 (4.7) | 43 (18.4) |  |
| T2 | | | | |  | 15 (23.1) | | 62 (19) | | | |  | |  | 6 (9.4) | 40 (17.1) |  |
| T3 | | | | |  | 36 (55.4) | | 140 (42.9) | | | |  | |  | 51 (79.7) | 126 (53.8) |  |
| T4 | | | | |  | - | | 5 (1.5) | | | |  | |  | 3 (4.7) | 12 (5.1) |  |
| Missing | | | | |  | - | | 1 (0.3) | | | |  | |  | - | - |  |
| **ypN stage** | | | | |  |  | |  | | | | 0.936 | |  |  |  | 0.264 |
| N0 | | | | |  | 33 (50.8) | | 179 (54.9) | | | |  | |  | 17 (26.6) | 77 (32.9) |  |
| N1 | | | | |  | 20 (30.8) | | 90 (27.6) | | | |  | |  | 17 (26.6) | 78 (33.3) |  |
| N2 | | | | |  | 7 (10.8) | | 34 (10.4) | | | |  | |  | 16 (25) | 46 (19.7) |  |
| N3 | | | | |  | 5 (7.7) | | 22 (6.7) | | | |  | |  | 33 (14.1) | 33 (14.1) |  |
| Missing | | | | |  | - | | 1 (0.3) | | | |  | |  | - | - |  |
| **ypM stage** | | | | |  |  | |  | | | | 0.119 | |  |  |  | 0.583 |
| M0 | | | | |  | 59 (90.8) | | 316 (96.9) | | | |  | |  | 58 (90.6) | 219 (93.6) |  |
| M1 | | | | |  | 5 (7.7) | | 8 (2.5) | | | |  | |  | 6 (9.4) | 15 (6.4) |  |
| Mx | | | | |  | 1 (1.5) | | 1 (0.3) | | | |  | |  | - | - |  |
| Missing | | | | |  | 0 (0) | | 1 (0.3) | | | |  | |  | - | - |  |
| **Mandard** | | | | |  |  | |  | | | | 0.204 | |  |  |  | 0.448 |
| TRG 1-2 | | | | |  | 28 (43.1) | | 153 (46.9) | | | |  | |  | 5 (7.8) | 30 (12.8) |  |
| TRG 3-5 | | | | |  | 37 (56.9) | | 161 (49.4) | | | |  | |  | 46 (71.9) | 151 (64.5) |  |
| Missing | | | | |  | - | | 12 (3.7) | | | |  | |  | 13 (20.3) | 53 (22.6) |  |
| **Resection margin** | | | | | |  | |  | | | | 0.670 | |  |  |  | **0.039** |
| R0 | | | | |  | 59 (90.8) | | 301 (92.3) | | | |  | |  | 45 (70.3) | 192 (82.1) |  |
| R1/R2 | | | | |  | 6 (9.2) | | 25 (7.7) | | | |  | |  | 19 (29.7) | 42 (17.9) |  |
| **Lymph node yield, median [IQR]** | | | | |  | 20 [10] | | 18 [10] | | | | 0.335 | |  | 22.5 [14] | 24 [15] | 0.470 |
| **Positive lymph nodes, median [IQR]** | | | | |  | 0 [2] | | 0 [2] | | | | 0.355 | |  | 4 [7] | 2 [5] | **0.004** |
| *nCRT* neoadjuvant chemoradiotherapy, *nCT* neoadjuvant chemotherapy, *SRC* signet ring cell, *TRG* tumour regression grade, *IQR* interquartile range; **bold** values are statistically significant (*p* < 0.05; two-sided) | | | | | | | | | | | | | | | | | |
|  | |  |  | | | |  | | | |  | |  |  |  |  |  |
|  |  | | |  | | | | |  |  |  |  |  |  |  |  |  |
|  |  | | |  | | | | |  |  |  |  |  |  |  |  |  |
|  |  | | |  | | | | |  |  |  |  |  |  |  |  |  |
|  | |  |  | | | |  | | | |  | |  |  |  |  |  |
